# Optimizing AI-assisted decision-making in mammography screening: focusing on uncertainties

**DOI:** 10.1186/s13244-026-02411-4

**Published:** 2026-10-13

**Authors:** Nasrin Ahmadinejad, Nahid Sadighi, Rassa Ghavami Modegh, Mehran Arab Ahmadi, Mohammadhosein Akhlaghpasand, Alireza Dizaji, Amin Rezaee, Maryam Rahmani, Arvin Arian, Fahimeh Zeinalkhani, Salome Maghsudlu, Saeedeh Shokri, Niloofar Pashaeifakhri, Hossein Ghanaati, Ali Sefidmouy, Mahdi Ghaznavi, Hamed Dashti, Hamid R. Rabiee, Masoumeh Gity

**Affiliations:** 1 Advanced Diagnosis and Interventional Radiology Research Center, Imam Khomeini Hospital, Tehran, Iran; 2 Department of Radiology, Tehran University of Medical Sciences, Tehran, Iran; 3 Department of Computer Engineering, Sharif University of Technology, Tehran, Iran; 4 Science and Technology Park of Sharif University of Technology, Tehran, Iran

**Keywords:** Artificial intelligence, Breast cancer screening, Mammography, Computer-aided detection system

## Abstract

**Objective:**

To evaluate an AI-based decision-support system for mammography interpretation, assessing its impact on diagnostic performance.

**Materials and methods:**

This retrospective study analyzed 130,838 mammograms from 35,709 patients with confirmed follow-up outcomes across multiple centers, using the Digital Database for Screening Mammography (DDSM) for external validation. Representing one of the largest datasets from the Middle East, it evaluates model generalizability in underrepresented populations. An AI super-model incorporated separate UNet-based networks for lesion segmentation, a high-resolution model (4096) for calcifications, a multi-resolution ensemble (4096, 2048, 1024) for macro-scale lesions, and a risk-assessment model for malignancy prediction. Radiologists interpreted images under five scenarios: unaided, AI-assisted, AI as the final decision-maker in uncertain cases, union of radiologist and AI decisions in uncertain cases, and standalone AI.

**Results:**

AI assistance significantly enhanced performance across all scenarios. Based on aggregated reader performance, AUC improved from 83.04% (95% CI: 79.8–86.3) unaided to 88.40% (95% CI: 85.6–91.2) in the union scenario (*p* < 0.001). Sensitivity increased from 54.8% (95% CI: 48.6–61.0) to 83.9% (95% CI: 79.3–88.4) (*p* < 0.001), and specificity rose from 83.6% (95% CI: 82.0–85.3) to 91.1% (95% CI: 89.8–92.4) (*p* < 0.001). Multi-resolution segmentation effectively detected calcifications and challenging lesions. DDSM validation confirmed model robustness. AI showed particular promise in aiding less-experienced radiologists in underserved areas to improve early cancer detection.

**Conclusions:**

AI in mammography interpretation improves diagnostic accuracy and reduces uncertainty, supporting early breast cancer detection and optimized screening workflows.

**Key Points:**

***Question*** Can an AI-based decision-support system improve the accuracy of mammography interpretation, reduce diagnostic uncertainty, and support less-experienced radiologists?

***Findings*** In this retrospective multicenter study, AI-assisted mammography improved sensitivity and specificity, increased cancer detection, and reduced recalls and false positives.

***Critical relevance statement*** AI integration in mammography enhances diagnostic accuracy, reduces unnecessary recalls, and supports more confident and efficient clinical decision-making, particularly by improving the performance of less-experienced radiologists in underserved and resource-limited settings where expert interpretation may be limited.

**Graphical Abstract:**

**Figure:**
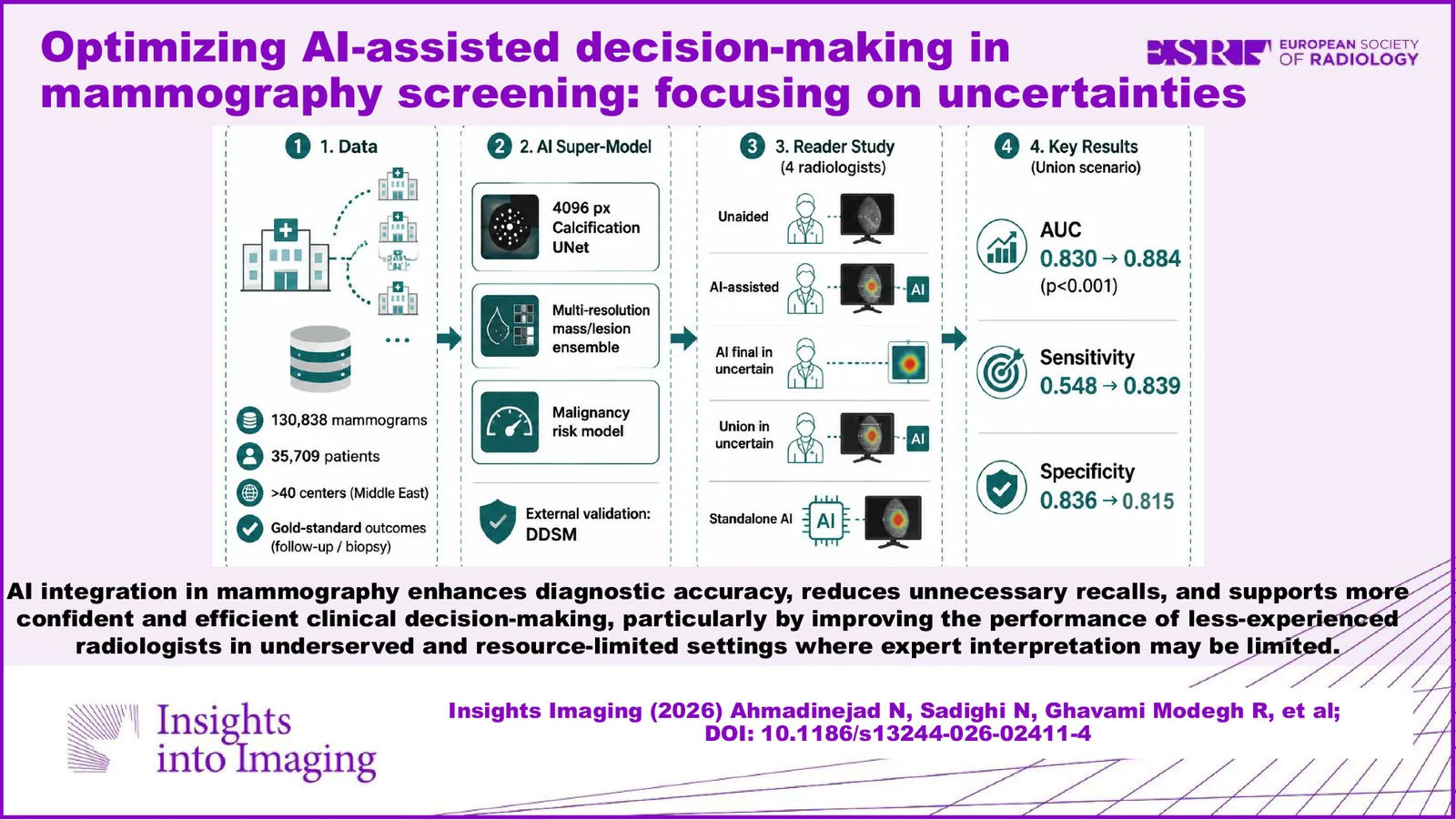

## Introduction

Breast cancer is the most common cause of cancer-related deaths in women, affecting 1 in 8 women and incurring a $16.5 billion economic burden in 2020 [1, 2]. Despite high survival rates (80–90% at 5 years with early diagnosis), breast cancer cases are rising, with 70% of the increase expected in low- and middle-income countries by 2030 [3, 4]. To support timely detection, the World Health Organization (WHO) recommends mammography screening programs [5]. These programs, while widely used, differ in methods (e.g., single reading in the U.S. vs. double reading in Europe), starting age (40–50 years), and screening intervals (annual to triennial) [6–9]. Mammography, the primary imaging tool, faces cost-effectiveness challenges due to false positives, overdiagnosis, and interval cancers [10, 11]. Increasing workload pressures and staffing shortages among radiologists and radiographers have prompted the exploration of alternative strategies, such as computer-aided detection (CAD), to improve efficiency and sustain breast cancer screening programs [12, 13].

More than five mammographic interpretation algorithms have been approved by the FDA, primarily as clinical decision-support tools to improve diagnostic accuracy and efficiency [14]. Machine learned-based CAD systems have shown performance equal to or better than human experts, providing independent case-level outputs for standard two-dimensional mammographic views, including mediolateral oblique and craniocaudal images [13, 15]. These algorithms are used for standalone CAD for automated interpretation, CADx to support clinical decision-making, and CADt to prioritize high-risk cases when set for high sensitivity [16]. These advancements could revolutionize breast cancer screening by enhancing diagnostic precision, optimizing resource allocation, and addressing challenges like radiologist shortages and growing workloads.

Research on traditional CAD systems for mammography has shown limited improvements in diagnostic performance, with sensitivity similar to double reading but higher recall rates, raising concerns about cost-effectiveness [17, 18]. These systems are limited by high false-positive rates, inconsistent reproducibility, long reading times, and a detection bias favoring calcifications over soft-tissue abnormalities and architectural distortions [19, 20]. These limitations arise from relying on handcrafted features derived from human annotations, which limit adaptability and precision. However, integrating them into clinical workflows is complex because they must meet a “clinically relevant threshold” without increasing recall rates, which could strain resources and necessitate human oversight for specificity management [14, 21]. While these advancements are promising, further research is needed to determine their clinical utility and optimize implementation.

This study developed an advanced AI model using UNet-style neural networks for lesion detection and a risk-assessment model to assist radiologists in mammogram interpretation. The model was trained on a mammogram dataset for lesion segmentation and risk assessment, and the study evaluated the integration of CAD systems into clinical workflows while addressing diagnostic uncertainties.

## Materials and methods

### Study design

This retrospective cohort study was approved by the Ethics Committee of Tehran University of Medical Sciences (IR.TUMS.IKHC.REC.1399.255) and conducted in accordance with the Declaration of Helsinki. All patients had previously provided written informed consent for the anonymized use of their data in research; therefore, no additional consent was required. The CLAIM checklist guidelines are provided in Supplementary Table S10.

**Fig. 1:**
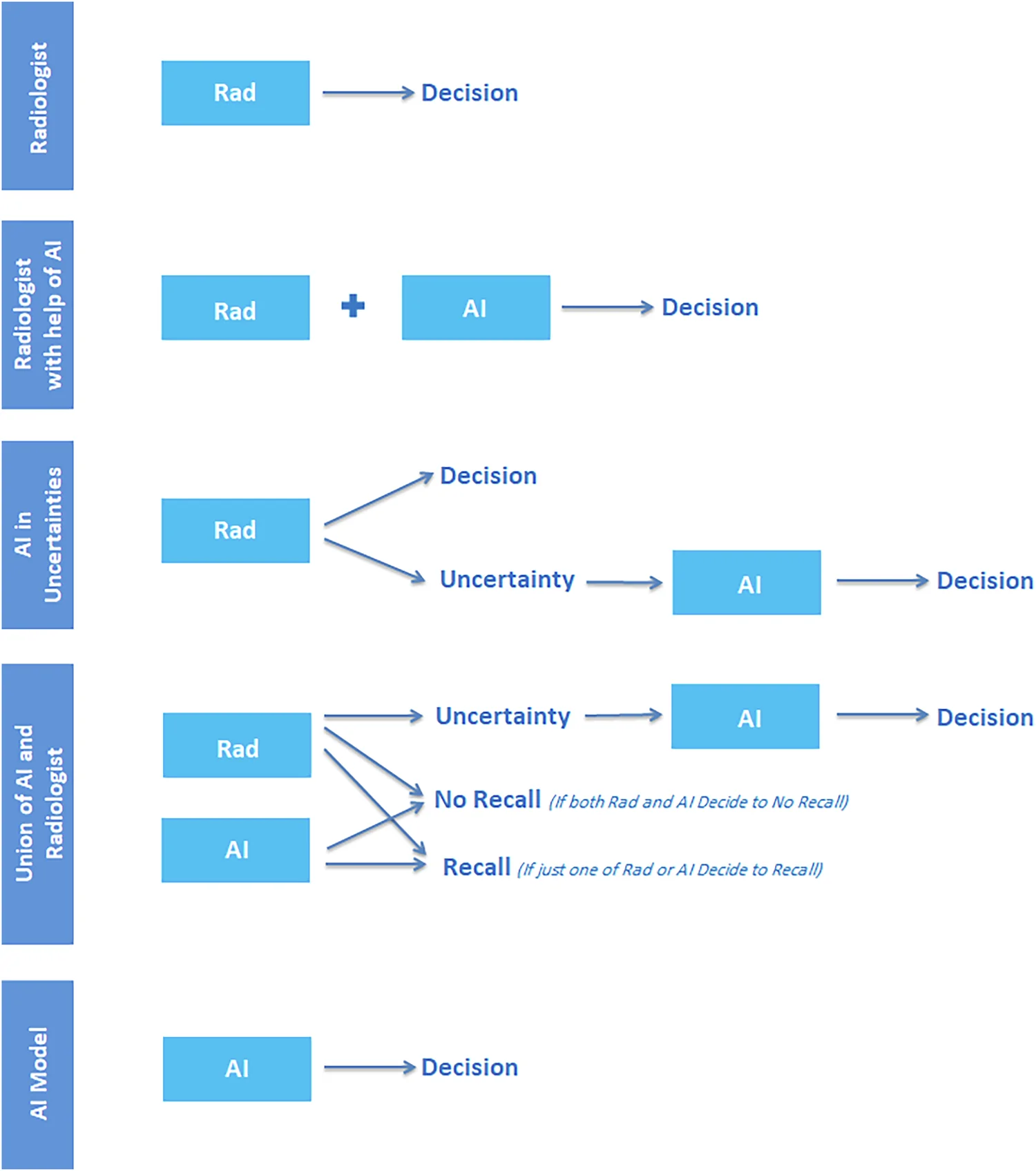
Integration of AI into screening workflow; overview of the five scenarios for integrating the AI system into the screening workflow: (1) traditional radiologist assessment, (2) adjunctive interpretation allowing radiologists to revise assessments based on AI input, (3) AI as the final decision-maker in uncertain cases, (4) a union scenario combining the highest risk predictions from AI and radiologists, and (5) AI functioning independently

The study aimed to develop an advanced AI super-model to assist radiologists in interpreting mammograms by identifying and analyzing lesions, including macro-scale lesions and calcifications, and assigning a risk score to each image. A comprehensive dataset of 130,838 high-quality screening mammography images, with complete follow-up data from May 2009 to December 2020, was collected from various academic and private hospitals and radiology centers in Iran. All images were full-field digital mammograms (FFDM) acquired using Hologic, Fujifilm, and Payamed devices. This study represents one of the largest datasets in this field from the Middle East to date, providing a valuable basis for evaluating model generalizability across underrepresented populations. To evaluate the model under different application scenarios (shown in Fig. 1), the CBIS-DDSM dataset [22, 23], a curated version of the Digital Database for Screening Mammography (DDSM) [24, 25], was incorporated as an external cohort. The curated dataset contains four standard-view mammograms from 1566 women, comprising 752 biopsy-proven malignant and 666 biopsy-proven benign cases. An additional 148 cases are labeled as benign based on expert radiologist assessment. Notably, the CBIS-DDSM dataset is reported to include validated ground-truth labels [22]. While normal cases have a documented 4-year follow-up, no specific follow-up duration has been reported for the 148 benign-without-callback cases [24]. CBIS-DDSM is one of the most widely adopted datasets in mammography CAD research; its use provides a robust external validation framework for the study.

The internal dataset was divided into training, validation, and test sets with 70%, 15%, and 15% allocations, respectively, ensuring a balanced distribution of breast compositions and BI-RADS scores. The DDSM dataset followed the original separation.

We also conducted a clinical study to evaluate the effectiveness of using a trained AI assistant for interpreting 2D digital mammograms. To this end, we randomly selected 600 screening cases comprising 2202 mammograms from the independent test set. These cases were confirmed by pathology, had at least 2 years of follow-up, and were not initially categorized as BI-RADS 3 by expert radiologists. The number of mammograms included in the clinical study was selected in accordance with those used in similar prior studies [26, 27]. Importantly, none of these cases were used in the training or validation phases of model development. The final dataset contained 120 positive and 480 negative examinations.

**Fig. 2:**
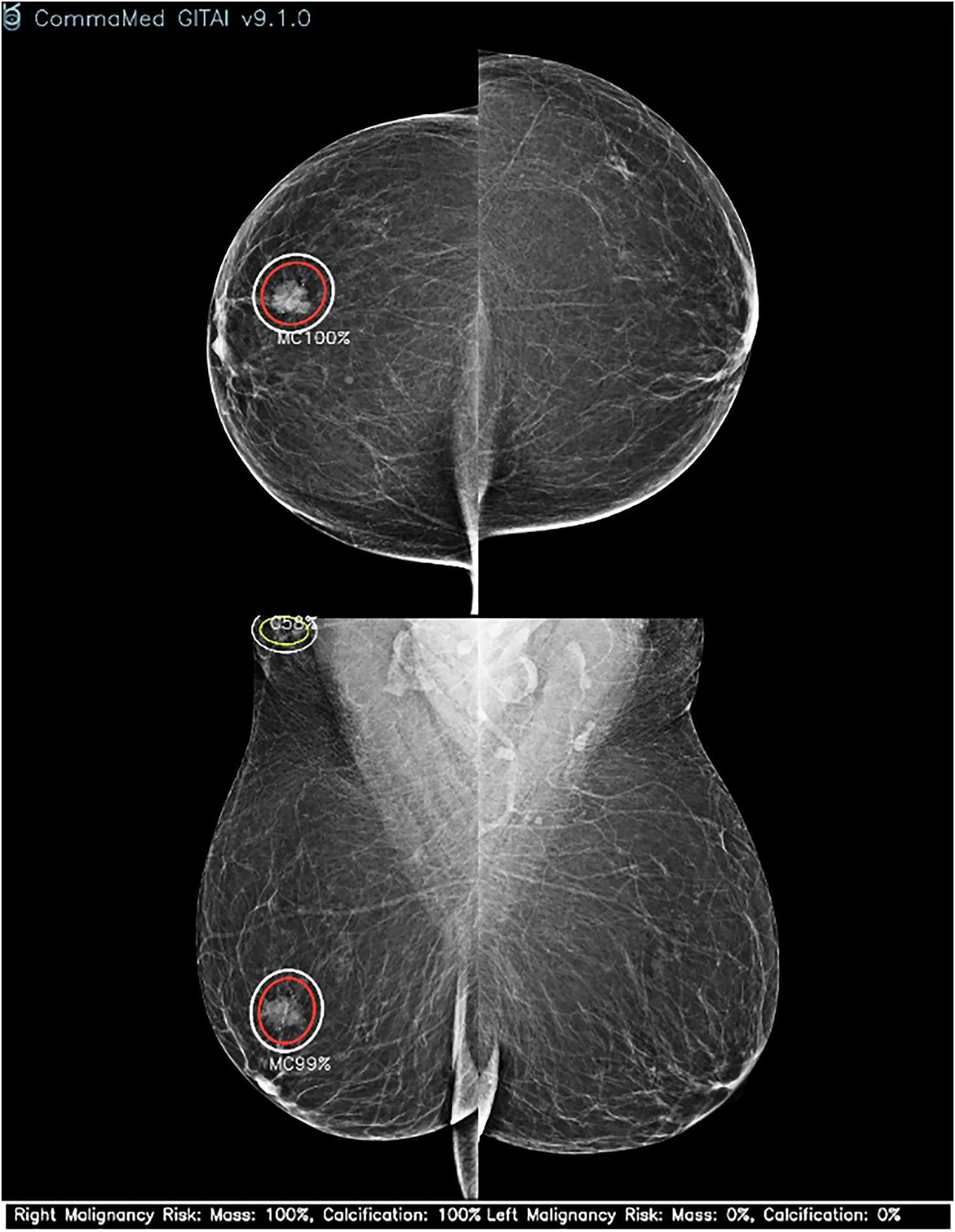
Example of an AI visualization interface. The AI system highlights lesion candidates with color-coded region-of-interest (ROI) overlays on mammographic images. A global malignancy risk score (0–100) is shown alongside the image. Radiologists first provided their unaided interpretation; AI overlays and the risk score were displayed only afterward during the AI-assisted session. This example demonstrates the visual format used in the reader study

Four junior radiologists with fewer than 6 years of experience in mammography participated in the study and independently interpreted the cases in a blinded reading setting. Readers 1–4 had 4, 3, 5, and 2 years of mammography experience, respectively. Each radiologist independently interpreted 300 cases with the same distribution of 60 positive and 240 negative cases. Readers received comprehensive instructions on the AI system. In the AI-assisted session, the AI identified lesions, estimated their malignancy risk, and presented them to the radiologist. A sample AI output, including the complete visualization interface, is shown in Fig. 2.

The AI assistant was employed in an adjunctive setting. In this approach, each mammogram was initially interpreted by a radiologist, and their preliminary interpretation was recorded. The AI assistant’s output was then presented, allowing the radiologist to revise their interpretation. The cases were ordered randomly for each reader.

To follow the scoring system used by radiologists in clinical practice, namely the Breast Imaging Reporting and Data System (BI-RADS), the radiologists assigned BI-RADS scores to each breast. Although the cases labeled BI-RADS 3 by the experts were excluded from the study, participants were permitted to assign BI-RADS 3 or BI-RADS 0 when they were uncertain about malignancy. BI-RADS 0 represents an “incomplete assessment” in 2D mammography, whereas BI-RADS 3 corresponds to a “probably benign” category requiring short-term follow-up. They were also asked to determine the lesion type and its anatomical location in both breasts using standard BI-RADS localization descriptors.

### Data preparation

Mammograms were extracted from the PACS of participating centers, and BI-RADS reports were retrieved from the HIS. The dataset, comprising images from multiple mammography devices, was anonymized to protect privacy. Two radiologists reviewed the images to identify and mark lesions. Notably, these radiologists had no overlap with the four radiologists who took part in the clinical study.

The DICOM images undergo a series of preprocessing steps: windowing parameters from the device header are applied, followed by normalization to the [0, 1] range. The images are padded with black pixels to make them square, then resized to 4096 × 4096 for input to the super-model.

### Model design

The super-model design incorporates separate models for lesion segmentation, with a specialized UNet-style Deep Neural Network (DNN) for segmenting calcification lesions. To accurately detect calcifications, including micro-calcifications, the input mammograms are processed at a high resolution of 4096.

The super-model uses three additional UNet-style DNNs to segment macro-scale lesions at varying resolutions (4096, 2048, 1024). For the 4096-resolution input, images are cropped into 512 × 512 pixel patches with a 256-pixel overlap. These patches are processed separately, and their outputs are combined to generate the final macro-segmentation.

**Fig. 3:**
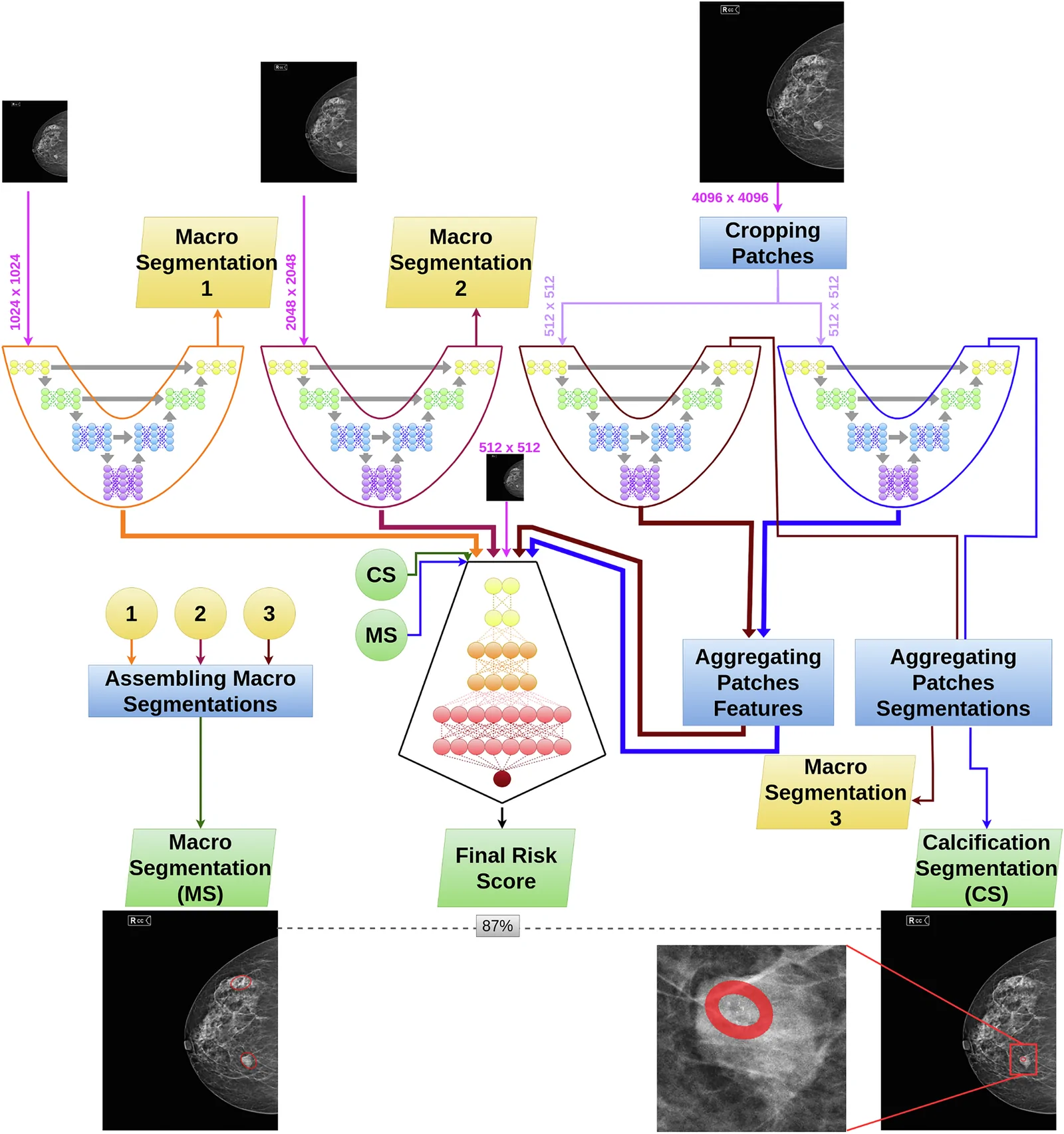
Development of the super-model; illustration of the architectures used in the super-model, each trained separately for specific tasks to optimize performance in mammographic interpretation

In the final stage, the outputs from the segmentation models and the original image are combined and processed by a DNN to calculate the overall risk score for the mammogram. Notably, all of the models are combinations of 2D Convolutions, Batch Norms, ReLU activations, and Skip Connections [28], Fully Connected Layers, Dropouts, and LAP [29] layers. The architecture is shown in Fig. 3.

### Model training

The super-model was developed in a stepwise approach: first, the segmentation models were trained and optimized, then frozen. Their outputs and embeddings were used as inputs to train the risk-assessment model, ensuring stable and improved performance.

#### Training segmentation models

Each segmentation model was trained independently using binary annotated lesion masks as ground truth. To address class imbalance in medical image segmentation, a pixel-wise Balanced Cross-Entropy loss function was used. This loss function accounts for the number of lesion pixels (N+) and non-lesion pixels (N−) in the training data. The function also incorporates the image width (W) and height (H), along with the probability that each pixel is associated with a lesion (Pijd), compared with the ground-truth label (GTijd), where the ground truth is 1 for lesion pixels and 0 for non-lesion pixels.

1 $${\sum}_{i,j\in \left[0,W\right]\times \left[0,H\right]}^{d\in \left[0,D\right]}\frac{1}{{N}^{+}}\left({{{\mathrm{ln}}}}{\left({P}_{{ij}}^{d}\right)}^{G{T}_{{ij}}^{d}}\right)+\frac{1}{{N}^{-}}\left({{{\mathrm{ln}}}}{\left(1-{P}_{{ij}}^{d}\right)}^{1-G{T}_{{ij}}^{d}}\right)$$

To further evaluate the model, the lesion capture rate (LCR) and false-positive rate (FPR) per image are calculated. The LCR (sensitivity) is used to measure how well the model detects lesions. At the same time, the FPR per Image reflects the model’s tendency to incorrectly classify non-lesion areas as lesions, which inversely relates to the model’s specificity. Each lesion is considered a true positive (TP) if its intersection with the ground truth mask is at least 50%; otherwise, it’s classified as a false positive (FP). These metrics together help to assess the model’s ability to accurately detect and localize lesions in mammograms, ensuring a balance between high lesion detection rates and minimizing false positives to improve the model’s overall diagnostic performance.

2 $${LCR}=\frac{{{TP}}_{{Lesions}}}{{N}_{{Lesions}}}$$

3 $${FPR}=\frac{{{FP}}_{{Lesions}}}{D}$$

The study focuses on optimizing a model for mammogram lesion detection using a modified F2 metric. This metric prioritizes lesion capture rate (LCR) over false-positive rate (FPR), addressing the imbalance in datasets in which most images contain no lesions. The approach ensures that the model detects as many lesions as possible while minimizing false positives.

4 $$F2=\frac{{2}^{2}+1}{\frac{{2}^{2}}{{LCR}}+\frac{1}{{Precision}{\prime} }}$$

5 $${{Precision}^{\prime} }=\frac{LCR}{{LCR}+{FPR}}$$

#### Training risk-assessment model

The risk-assessment model aims to differentiate images with malignant lesions from those without. Ideally, training would use pathology-proven cancer cases as positive examples and benign cases (or those with several years of follow-up) as negative examples. However, the limited number of positive cases could lead to overfitting; therefore, expert assessments from radiologists with over 25 years of experience were used as positive cases during the training phase. BI-RADS scores of 4, 4a, 4b, 4c, 5, and 6 were classified as positive, while BI-RADS scores of 1 and 2 were considered negative. Cases with BIRADS scores of 3 and 0 were excluded from training. Despite this approach, which aimed to enhance training by including more cases, the model was ultimately evaluated on a test set comprising only pathology-proven cancer cases and negative cases with at least 2 years of follow-up to ensure the validity of the reported performance metrics. The final classifier was trained using a balanced cross-entropy loss function.

We used Youden’s J statistic to determine the operating cut-off for classifying AI risk scores as either suspicious or non-suspicious, utilizing the validation set. This method selects the optimal cut-off by maximizing the sum of sensitivity and specificity, thereby balancing the rates of false-positive and false-negative. The final model’s performance is evaluated using sensitivity, specificity, and AUC. The AUC metric was used to select the final model due to its robustness in label distribution.

#### Optimization

All models are trained using the PyTorch framework [30] with the Adam [31] optimizer with a learning rate of 1E-4, on batches processed on a Quadro 8000 GPU. To improve model generalization and prevent overfitting, the study employs image augmentation techniques, including cropping, flipping, and contrast adjustment, during training. Additionally, during training epochs, each FFDM image was randomly presented either in its original form or as a transformed version using randomized linear and logarithmic intensity augmentation to produce images more visually similar to DDSM or digitized CR images, thereby ensuring that the final model generalizes to both standard FFDM and digitized DDSM images. This approach enables the model to learn representations that are robust to differences in image appearance between FFDM and digitized film mammograms, without requiring separate preprocessing or explicit domain adaptation techniques. Multiple models with varying hyperparameters are evaluated, and the best-performing model on the validation set is selected as the final model. This ensures that the model is robust and generalizes well to new data.

#### Different scenarios for the adoption of an AI assistant

##### Unassisted:

This is the base scenario in which the radiologist’s pre-review score for the AI interpretation of the case was recorded and used to assess the other assisted scenarios.

##### AI-assisted:

In this scenario, the radiologist’s revised score, recorded after reviewing the AI interpretation, is considered the final risk score.

##### AI in uncertain cases:

In this scenario, for cases the radiologist had labeled as certain, the radiologist’s post-review score of the AI interpretation was used as the final score. In the cases marked as uncertain, the AI system’s interpretation was used as the deciding factor for determining the presence or absence of malignancy.

##### Union:

In this consensus, the most severe decision assigned by the radiologist or the AI assistant was considered the final decision for the case. In cases where the AI detected a lesion with a radiologist’s BI-RADS 1 label, the lesion was considered not clinically reportable, and the final decision was revised to BI-RADS 1.

#### Statistical analysis

To evaluate the statistical significance of improvements in radiologists’ performance across each AI-assisted interpretation scenario, *p*-values were calculated using paired *z*-tests and *t*-tests, with comparisons against the baseline performance of unassisted radiologists. Differences were considered statistically significant at *p* < 0.05.

## Results

### Study population

The study analyzed a population of 130,838 individuals aged 18–93 years, with a mean age of 50.4 years (SD = 9.1). The majority of participants were female (*n* = 130,252), with a small number of males (*n* = 134) and cases with unknown sex (*n* = 452). Breast composition was distributed across categories A (*n* = 11,523), B (*n* = 43,324), C (*n* = 56,228), and D (*n* = 10,535), while 9228 cases had unknown composition (Table 1).

**Table 1:** Population characteristics: demographic and clinical characteristics of the study population, including age, sex, breast composition, and biopsy-proven malignant images

Population characteristics		*N* (%)/mean (SD)
Age		50.4 (9.1)
Sex	Female	130,252
	Male	134
	Unknown	452
Breast composition	A	11,523
	B	43,324
	C	56,228
	D	10,535
	Unknown	9228
Biopsy-proven malignant images		3361

Based on BI-RADS classification following diagnostic imaging, 6997 cases were categorized as BI-RADS 4+ (indicating suspicion of malignancy), 84,382 as BI-RADS 1 or 2 (benign findings), and 39,459 as BI-RADS 0 or 3 (requiring additional imaging or short-term follow-up). Additionally, 3361 images were confirmed as malignant through biopsy (Table 1).

### Super-model performance

The super-model has been evaluated from three primary perspectives: the performance of the calcification segmentation model, the macro-segmentation model, and the risk-assessment model. The calcification segmentation model demonstrated a lesion capture rate (LCR) of 92.99%, a false-positive rate (FPR) of 0.1 per image, and an F2 score of 92.43%, highlighting its exceptional precision and reliability in detecting calcifications. The macro-segmentation model, designed to identify larger-scale lesions, achieved an LCR of 90.00%, an FPR of 0.89 per image, and an F2 score of 77.71%, indicating effective performance despite a slightly higher false-positive rate than the calcification model (Table 2).

**Table 2:** Performance metrics of the trained models: lesion capture rate (LCR), false-positive rate (FPR), and F2 score for segmentation models, and sensitivity, specificity, and area under the curve (AUC) for the risk-assessment model

Trained models	Performance	
Calcification segmentation model	LCR: 92.99% (95% CI: 85.0–100.0)	
	FPR: 0.1/Image (95% CI: 0.09–0.11)	
	F2: 92.43% (95% CI: 85.89–98.05)	
Macro-segmentation model	LCR: 90.00% (95% CI: 82.93–95.7)	
	FPR: 0.89/Image (95% CI: 0.86–0.92)	
	F2: 77.71% (95% CI: 72.44–81.87)	
Risk-assessment model	Versus gold standard pathology or follow-up	Versus expert labels
	Sensitivity: 82% (78.5–85.5%)	Sensitivity: 91.98% (90.1–93.8%)
	Specificity: 74% (72.9–75.1%)	Specificity: 73.73% (72.6–74.8%)
	AUC: 88 (85.9–90.1)	AUC: 92 (90.7–93.3)

The risk-assessment model was evaluated using two benchmarks. Against pathology-proven labels and negative cases with at least 2 years of follow-up, it achieved a sensitivity of 82% (95% CI 78.5–85.5), specificity of 74% (95% CI 72.9–75.1), and an AUC of 88% (95% CI 85.9–90.1). Compared with expert recall decisions for biopsies, it demonstrated a sensitivity of 91.98% (95% CI 90.1–93.8), a specificity of 73.73% (95% CI 72.6–74.8), and an AUC of 92% (95% CI 90.7–93.3), indicating strong agreement with expert assessments. The lesion capture rate for segmentation models was defined as the proportion of lesions recalled for biopsy by expert radiologists. Additionally, the risk assessment scores were converted into binary outcomes (recalled or not) using a threshold of 65, which is determined by maximizing Youden’s J statistic on the validation dataset. These metrics, detailed in Table 2, show the model’s potential to improve lesion detection and risk assessment in clinical workflows.

### Performance AI super-model in different scenarios for the application

The primary AI model was evaluated through four distinct methods, integrated into a workflow to assist decision-making. The performance of various scenarios was assessed for each radiologist individually and in aggregate. AI integration significantly enhanced diagnostic performance across multiple metrics. Based on aggregated reader performance, unaided radiologists achieved an AUC of 83.04 (95% CI 79.8–86.3), which increased to 83.96 (95% CI 80.8–87.1) with AI assistance and further improved to 84.33 (95% CI 81.2–87.5) in the AI-in-uncertain-cases scenario (I/U). The union model achieved the highest AUC of 88.40 (95% CI 85.6–91.2), demonstrating the benefits of collaborative decision-making. AI alone achieved an AUC of 85.86 (95% CI 81.6–90.1), comparable to that of unaided radiologists, but it was surpassed when combined with radiologist input. All scenarios showed statistically significant improvements (*p*-values < 0.001). Uncertainty analysis revealed an AUC of 88.14 (95% CI 84.2–92.1) in ambiguous cases, highlighting AI’s strong performance even in uncertain diagnostic contexts. These findings, along with per-reader metrics, are detailed in Table 3.

**Table 3:** AUC of single radiologists for recall evaluation before (unaided) and after (aided) presenting AI’s interpretations, replacing AI’s opinion in uncertainty (AI in I/U), Union of radiologist and AI, and AI’s scores as a standalone reader

	Rad (unaided)	Rad (aided)	Rad + AI in I/U	Rad + AI (union)	AI	Uncertainty
Reader1	81.28	82.10	82.30	88.22	88.08	8.5
Reader2	84.66	85.63	86.09	90.65	88.77	10.8
Reader3	83.78	84.55	84.96	87.00	86.15	13.9
Reader4	82.99	84.96	85.85	88.71	89.94	22.6
All	83.04	83.96	84.33	88.40	88.14	13.7
95% CI	79.8–86.3	80.8–87.1	81.2–87.5	85.6–91.2	84.2–92.1	
*p*-value	-	< 0.001	< 0.001	< 0.001	< 0.001	

*p*-values demonstrate the significance of change between each scenario and unaided radiologist, indicating AI has enhanced the performance significantly in all scenarios. For all readers, the highest performance has been achieved by the union of AI and the radiologist

Evaluation of sensitivity and specificity across scenarios demonstrated significant improvements in radiological performance with AI assistance. Without considering uncertainty, based on aggregated reader performance, the sensitivity of unaided radiologists increased from 54.8% (95% CI 48.6–61.0) to 59.0% (95% CI 52.7–65.0) with AI aid and rose significantly to 70.5% (95% CI 64.9–76.2) in the AI-assisted I/U scenario, peaking at 83.9% (95% CI 79.3–88.4) in the union model (*p* < 0.001). Specificity also performed strongly, with the union model achieving 91.1% (95% CI 89.8–92.4), significantly higher than the 83.6% (95% CI 82.0–85.6) achieved by unaided radiologists (*p* < 0.001). When uncertainty was included, sensitivity increased from 67.3% (95% CI 61.5–73.2) (unaided) to 83.9% (95% CI 79.3–88.4) in the union scenario, and specificity peaked at 91.1% (95% CI 89.8–92.4). The *p*-values across all scenarios highlighted the statistical significance of these improvements, confirming AI’s role in enhancing diagnostic accuracy and reliability. These results are detailed in Table 4. Per-reader performance metrics are provided in Supplementary Tables 3–6. In addition, PPV, NPV, and recall rates were calculated across different scenarios, and the results are reported in Tables S7–S9.

**Table 4:** Sensitivity and specificity of all readers before (unaided) and after (aided) presenting AI’s interpretations, replacing AI’s opinion in uncertainty (AI in I/U), Union of radiologists, and AI as a standalone reader

	Sensitivity					Specificity				
	Rad (unaided)	Rad (aided)	Rad + AI (in I/U)	Rad + AI (Union)	AI	Rad (unaided)	Rad (aided)	Rad + AI (in I/U)	Rad + AI (Union)	AI
All—without uncertainty	54.8	59.0	70.5	83.9	80.6	83.6	82.4	91.1	81.5	75.4
95% CI	48.6–61.0	52.7–65.0	64.9–76.2	79.3–88.4	73.7–87.6	82.0–85.3	80.8–84.1	89.8–92.4	79.8–83.3	72.7–78.1
*p*-value	-	0.01	< 0.001	< 0.001	< 0.001	-	0.02	< 0.001	0.24	0.02
All—with uncertainty	67.3	70.5	70.5	83.9	80.6	87.3	86.5	91.1	81.5	75.4
95% CI	61.5–73.2	64.9–76.2	64.9–76.2	79.3–88.4	73.7–87.6	85.8–88.7	85.0–88.0	89.8–92.4	79.8–83.3	72.7–78.1
*p*-value	-	0.04	0.1	< 0.001	< 0.001	-	0.01	< 0.001	< 0.001	< 0.001

The values are calculated with and without considering the incomplete assessment or uncertain cases of BIRADS0 and BIRADS3. *p*-values demonstrate the significance of change between each scenario and unaided radiologist. Although the AI in I/U scenario has significantly enhanced both sensitivity and specificity, the union scenario has led to a drastic enhancement in sensitivity without harming specificity much

### Recall assessment

**Fig. 4:**
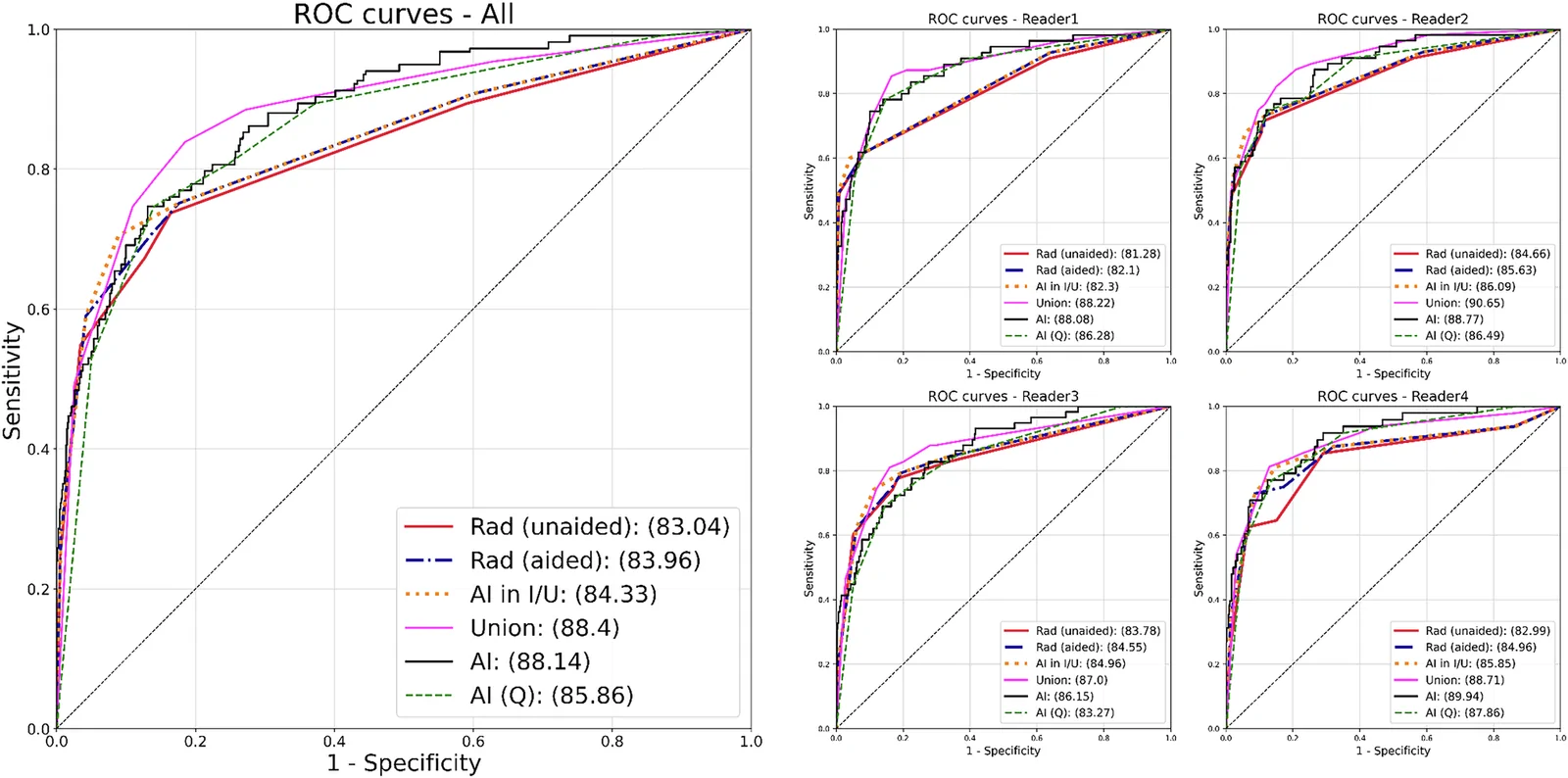
ROC curves for recall evaluation of the radiologists before (unaided) and after (aided) presenting AI’s interpretations, replacing AI’s opinion in uncertainties (AI in I/U), Union of radiologist and AI, and AI’s raw scores as a standalone reader. The curves are plotted for all readers and each of the four readers separately. Across all plots, the union has achieved the highest performance, underscoring the need for cooperation between radiologists and AI for efficient decision-making

We computed ROC curves using radiologists’ pre- and post-presentation AI interpretations. The ROC curves for unaided radiologists, AI as a standalone reader, and the three scenarios are shown in Fig. 4. It is evident that the ROC curves for radiologists in all three scenarios improved compared to the unaided case. The best curve is from the Union scenario, which outperformed the others, even AI alone, particularly in the decision-making interval where sensitivity and specificity exceed 60%. AUC scores and the significance of improvements compared to unaided radiologists are presented in Table 3. All three scenarios significantly improved radiologists’ performance (*p*-value < 0.001), with the union scenario achieving the most significant improvement (5.37%, *p*-value < 0.001). This scenario achieved the highest AUC across all readers, indicating that collaboration between radiologists and AI yields the most efficient decision-making.

### Lesion detection assessment

For further evaluation, we calculated the number of additions for each lesion type by comparing radiologists’ reports before and after the AI’s interpretations were presented. The results are presented in Table 5. It is observed that AI significantly influences the detection of missing lesions across all four lesion types (*p*-values ≤ 0.04). The highest addition rate is for calcifications, which are the smallest and easiest to miss lesions (0.0242, *p*-value ≤ 1E-4).

**Table 5:** The rate of additions per breast for each type of lesion after presenting AI’s interpretations

	Mass	Calcification	Distortion	Asymmetry
Number of added lesions of the type after revision per breast	0.0114	0.0242	0.0143	0.0193
*p*-value	1.5E-5	1.0E-4	9.3E-4	4.1E-3

The *p*-values represent the significance of addition rates, which indicate AI has been significantly effective in refining all four main types of breast lesions

## Discussion

The study evaluated an advanced AI model to detect lesions and assist radiologists in interpreting mammograms for breast cancer detection. The AI system was tested across five scenarios, including traditional radiologist assessment and different AI integrations. Based on aggregated reader performance, all three modes of AI adoption led to improved performance. The AUC of unassisted readers increased from 83.04 (95% CI 79.8–86.3) to 83.96 (95% CI 80.8–87.1), 84.33 (95% CI 81.2–87.5), and 88.40 (95% CI 85.6–91.2) in the AI-assisted, AI-in-uncertain-cases, and union scenarios, respectively. While the highest AUC was achieved in the union scenario, this improvement comes with trade-offs: the recall rate rose from 18.89% (95% CI 17.3–20.5) for unassisted radiologists to 25.84% (95% CI 24.0–27.7), and the PPV declined from 40.14% (95% CI 35.4–44.9) to 36.56% (95% CI 31.9–41.2). Given that this scenario also achieved the highest sensitivity—83.9% (95% CI 79.3–88.4)—and consequently the lowest rate of missed cancers, it may be suitable for settings where the cost of patient recall is low but the consequences of missed cancers are high. Conversely, the scenario in which AI judgments were incorporated only when the radiologist was uncertain yielded the highest specificity (91.1%, 95% CI 89.8–92.4), while still improving sensitivity (70.5%, 95% CI 64.9–76.2) compared with unassisted readings. As a result, the recall rate decreased to 15.85% (95% CI 14.3–17.4), and the PPV increased to 50.14% (95% CI 45.3–54.9), making this the most consistently beneficial strategy, with improvements across all evaluated performance metrics, and particularly advantageous in settings where recalling patients is costly. AI also helped reduce missed lesions, particularly calcifications, underscoring its potential to improve diagnostic precision, especially in resource-limited environments.

Researchers have recently paid significant attention to the application of AI systems for mammogram interpretation. Numerous studies have investigated the efficacy of AI as a computer-aided detection (CAD) system. Rodríguez-Ruiz et al [32] reported a notable two-percent increase in the average AUC following the use of an AI system, while Pacile et al [27] observed a 2.8% improvement. Similarly, FDA-conducted experiments demonstrated that Siemens’ Transpara and Hologic’s Genius AI Detection increased radiologists’ AUC from 83.3% to 86.3% and from 79.4% to 84.0%, respectively [33, 34]. Furthermore, LUNIT INSIGHT MMG, used as an adjunctive CAD system, increased the AUC from 75.4% to 80.5% [35]. These findings align with our study, which demonstrated a 1% to 5.4% improvement in AUC across different scenarios. The most minor improvement was observed in the free-adoption scenario, consistent with results from studies other than the FDA’s. In contrast, the most significant improvement occurred in the union scenario, consistent with FDA findings. Although some studies have focused on comparing AI as a standalone reader against radiologists [32, 36], others have highlighted that the optimal performance is achieved by combining AI with radiologist interpretations [37–39]. These results are consistent with our findings, which show that the most significant diagnostic accuracy was achieved in a combination scenario.

Previous studies have demonstrated that integrating AI systems as CAD tools can significantly enhance radiologists’ ability to detect cancers presented as masses, distortions, or asymmetries, particularly in challenging cases such as early-stage, node-negative invasive cancers or cancers in mammographically dense breasts [40]. These findings are particularly promising given the increased difficulty of detecting cancer under challenging conditions. In our study, we evaluated the impact of AI assistance on the detection of four primary lesion types: mass, distortion, calcification, and asymmetry. The results showed that the AI-CAD system significantly reduced missed lesion rates across all categories. The most notable improvement was for calcifications, the smallest and most difficult lesions to detect, which often require extensive zooming. These findings highlight AI’s potential to reduce missed cancers by identifying undetected lesions, thereby improving diagnostic accuracy.

This study simulated a realistic clinical workflow by allowing radiologists to use an “uncertain” label. It compared union and I/U scenarios, showing that AI alerts significantly improved diagnostic accuracy. The union scenario increased sensitivity while maintaining high specificity, effectively reducing missed cancer diagnoses. The I/U scenario also improved sensitivity and specificity. However, the aided radiologist scenario showed the least improvement, suggesting that AI risk scores alone don’t fully gain radiologists’ trust. The findings highlight the importance of integrating AI thoughtfully into clinical workflows for maximum effectiveness and acceptance.

Our findings show that AI can significantly improve radiologists’ performance, particularly in sensitivity, when interpreting 2D digital mammography images. AI is especially beneficial for less-experienced radiologists (2–5 years of experience in reading mammography). This highlights the potential role of AI in supporting mammography interpretation in settings with limited availability of experienced radiologists. In these contexts, AI support can reduce diagnostic uncertainty and increase the cancer detection rate. Moreover, this study represents one of the largest datasets in this field from the Middle East to date, providing a valuable basis for evaluating model generalizability across underrepresented populations.

This study has limitations, including a small number of radiologists and a retrospective design, which affect the robustness and generalizability of the findings. It also lacked an evaluation of AI’s impact on final recall rates, necessitating larger prospective studies to draw more reliable conclusions. Future research should address these limitations by including more diverse cohorts and real-world scenarios to validate and expand on the findings. In addition, the study cohort may not be representative of those in population-based screening programs, and prevalence was not stratified by subgroup. Performance was not analyzed by breast density, equipment vendor, or patient age, all of which may influence AI and reader performance. Finally, although recall rate, PPV, and NPV were reported, the study was not powered to draw definitive conclusions regarding these workflow-relevant metrics. Larger, prospective, and multicenter studies incorporating diverse populations, standardized pathology-based outcomes, detailed workflow measurements, and subgroup analyses are warranted to validate and extend these findings.

## Conclusion

This study demonstrates the significant potential of AI to enhance mammography screening by improving diagnostic accuracy, sensitivity, and specificity, particularly for less-experienced radiologists and in challenging cases. The best results were achieved through collaboration between AI and radiologists, highlighting the value of integrating AI into clinical workflows. While limitations exist, the findings underscore AI’s capacity to reduce missed diagnoses and improve early cancer detection, warranting further research across diverse healthcare settings.

## Supplementary information

ELECTRONIC SUPPLEMENTARY MATERIAL

## Data Availability

The datasets used and/or analyzed during the current study are available from the corresponding authors upon reasonable request.

## Abbreviations

AI: Artificial intelligence
AUC: Area under the curve
BIRADS: Breast Imaging Reporting and Data System
CAD: Computer-aided detection
DDSM: Digital Database for Screening Mammography
FPR: False-positive rate
LCR: Lesion capture rate
