# Supplementary material for: Optimizing AI-assisted decision-making in mammography screening: focusing on uncertainties
Source: Insights Imaging. 2026 Oct 13;17:252. doi: 10.1186/s13244-026-02411-4 (PMC13661836; doi:10.1186/s13244-026-02411-4)
Supplement: Supplementary file 1 [file 13244_2026_2411_MOESM1_ESM.pdf]

Optimizing AI-Assisted Decision-Making in Mammography Screening: Focusing on Uncertainties

ELECTRONIC SUPPLEMENTARY MATERIAL

Supplementary Table S1. Count-based confusion matrices per scenario, showing the sum of all readers' interpretations, for per-breast analysis of the clinical trial involving 600 cases (4 bilateral cancers, 116 unilateral cancers, and 480 non-cancer cases), resulting in a total of 124 positive breasts and 977 negative breasts with known follow-up. Each case was independently interpreted by two participating radiologists.

	GT	Assigned Label				Total
		Negative	Positive	B0	B3	
Unassisted Radiologist	Negative	1634	61	188	71	1954
	Positive	63	136	31	18	248
Assisted Radiologist	Negative	1611	75	189	79	1954
	Positive	60	146	29	13	248
Rad + AI (I/U)	Negative	1780	174	0	0	1954
	Positive	73	175	0	0	248
Rad + AI (Union)	Negative	1593	361	0	0	1954
	Positive	40	208	0	0	248
AI	Negative	1474	480	0	0	1954
	Positive	48	200	0	0	248

Supplementary Table S2. Per-reader, per-scenario AUCs with 95% confidence intervals, alongside paired z-tests and paired t-tests to assess the significance of results compared to the baseline of unassisted radiologists. The results indicate significant performance improvements across all adoption scenarios, with the largest improvement observed in the "Union" adoption scenario.

AUC	**Rad (unaided)**	**Rad (aided)**	**Rad + AI in I/U**	**Rad + AI (union)**	**AI**
**Reader1**	81.28 (74.6–87.9)	82.1 (75.5–88.7)	82.3 (75.8–88.8)	88.22 (82.6–93.8)	88.08 (82.5–93.7)
**Reader2**	84.66 (78.5–90.8)	85.63 (79.6–91.7)	86.09 (80.1–92.0)	90.65 (85.6–95.7)	88.77 (83.3–94.2)
**Reader3**	83.78 (77.5–90.1)	84.55 (78.3–90.8)	84.96 (78.8–91.1)	87 (81.2–92.8)	86.15 (80.2–92.1)
**Reader4**	82.99 (76.6–89.4)	84.96 (78.8–91.1)	85.85 (79.8–91.8)	88.71 (83.2–94.2)	89.94 (84.7–95.2)
**All**	83.04 (79.8–86.3)	83.96 (80.8–87.1)	84.33 (81.2–87.5)	88.4 (85.6–91.2)	88.14 (84.2–92.1)
**Paired T-Test p-value**		0.0139	0.0154	0.0031	0.0099
**Paired Z-Test p-value**		0.0000	0.0001	0.0000	0.0000

Supplementary Table S3. Per-reader, per-scenario sensitivities with 95% confidence intervals, alongside paired z-tests and paired t-tests to assess the significance of results compared to the baseline of unassisted radiologists.

	**Rad (unaided)**	**Rad (aided)**	**Rad + AI in I/U**	**Rad + AI (union)**	**AI**
**Reader1**	64.52 (52.6–76.4)	66.13 (54.3–77.9)	64.52 (52.6–76.4)	87.1 (78.8–95.4)	85.48 (76.7–94.2)
**Reader2**	67.74 (56.1–79.4)	69.35 (57.9–80.8)	67.74 (56.1–79.4)	82.26 (72.8–91.8)	79.03 (68.9–89.2)
**Reader3**	70.97 (59.7–82.3)	74.19 (63.3–85.1)	72.58 (61.5–83.7)	85.48 (76.7–94.2)	79.03 (68.9–89.2)
**Reader4**	66.13 (54.3–77.9)	72.58 (61.5–83.7)	77.42 (67.0–87.8)	80.65 (70.8–90.5)	79.03 (68.9–89.2)
**All**	67.3 (61.5–73.1)	70.5 (64.8–76.2)	70.5 (64.8–76.2)	83.9 (79.3–88.5)	80.6 (73.6–87.6)
**Paired Z-Test p-value**	-	0.0024	0.1174	0.0000	0.0000
**Paired T-Test p-value**	-	0.0332	0.1602	0.0019	0.0084

Supplementary Table S4. Per-reader, per-scenario sensitivities, accounting only the certain scores, with 95% confidence intervals, alongside paired z-tests and paired t-tests to assess the significance of results compared to the baseline of unassisted radiologists.

	Rad (unaided)	Rad (aided)	Rad + AI in I/U	Rad + AI (union)	AI
**Reader1**	54.84 (42.5–67.2)	56.45 (44.1–68.8)	64.52 (52.6–76.4)	87.1 (78.8–95.4)	85.48 (76.7–94.2)
**Reader2**	46.77 (34.3–59.2)	51.61 (39.2–64.0)	67.74 (56.1–79.4)	82.26 (72.8–91.8)	79.03 (68.9–89.2)
**Reader3**	59.68 (47.5–71.9)	61.29 (49.2–73.4)	72.58 (61.5–83.7)	85.48 (76.7–94.2)	79.03 (68.9–89.2)
**Reader4**	58.06 (45.8–70.3)	66.13 (54.3–77.9)	77.42 (67.0–87.8)	80.65 (70.8–90.5)	79.03 (68.9–89.2)
**All**	54.8 (48.6–61.0)	59 (52.9–65.1)	70.5 (64.8–76.2)	83.9 (79.3–88.5)	80.6 (73.6–87.6)
**Paired Z-Test p-value**	-	0.0046	0.0000	0.0000	0.0000
**Paired T-Test p-value**	-	0.0399	0.0049	0.0011	0.0022

Supplementary Table S5. Per-reader, per-scenario specificities with 95% confidence intervals, alongside paired z-tests and paired t-tests to assess the significance of results compared to the baseline of unassisted radiologists.

	Rad (unaided)	Rad (aided)	Rad + AI in I/U	Rad + AI (union)	AI
**Reader1**	91.48 (89.0–93.9)	91.28 (88.8–93.8)	95.74 (94.0–97.5)	83.57 (80.3–86.8)	74.65 (70.8–78.5)
**Reader2**	89.41 (86.7–92.1)	88.8 (86.0–91.6)	93.89 (91.8–96.0)	84.52 (81.3–87.7)	76.37 (72.6–80.1)
**Reader3**	82.5 (79.1–85.9)	81.88 (78.4–85.3)	88.54 (85.7–91.4)	80 (76.4–83.6)	75.21 (71.3–79.1)
**Reader4**	85.51 (82.4–88.6)	83.88 (80.6–87.1)	86.12 (83.1–89.2)	77.96 (74.3–81.6)	75.51 (71.7–79.3)
**All**	87.3 (85.8–88.8)	86.5 (85.0–88.0)	91.1 (89.8–92.4)	81.5 (79.8–83.2)	75.4 (72.7–78.1)
**Paired Z-Test p-value**	-	0.006	0.0004	0.0000	0.0000
**Paired T-Test p-value**	-	0.0434	0.0221	0.0101	0.0052

Supplementary Table S6. Per-reader, per-scenario sensitivities with 95% confidence intervals, accounting only the certain scores, alongside paired z-tests and paired t-tests to assess the significance of results compared to the baseline of unassisted radiologists.

	**Rad (unaided)**	**Rad (aided)**	**Rad + AI in I/U**	**Rad + AI (union)**	**AI**
**Reader1**	91.08 (88.6–93.6)	90.87 (88.3–93.4)	95.74 (94.0–97.5)	83.57 (80.3–86.8)	74.65 (70.8–78.5)
**Reader2**	89.41 (86.7–92.1)	88.59 (85.8–91.4)	93.89 (91.8–96.0)	84.52 (81.3–87.7)	76.37 (72.6–80.1)
**Reader3**	81.46 (78.0–84.9)	80.42 (76.9–84.0)	88.54 (85.7–91.4)	80 (76.4–83.6)	75.21 (71.3–79.1)
**Reader4**	72.45 (68.5–76.4)	69.8 (65.7–73.9)	86.12 (83.1–89.2)	77.96 (74.3–81.6)	75.51 (71.7–79.3)
**All**	83.6 (82.0–85.2)	82.4 (80.7–84.1)	91.1 (89.8–92.4)	81.5 (79.8–83.2)	75.4 (72.7–78.1)
**Paired Z-Test p-value**	-	0.0117	0.0003	0.2295	0.0288
**Paired T-Test p-value**	-	0.0541	0.0201	0.2564	0.0769

Supplementary Table S7. Per-reader, per-scenario PPVs with 95% confidence intervals, alongside paired z-tests and paired t-tests to assess the significance of results compared to the baseline of unassisted radiologists.

PPV	**Rad (unaided)**	**Rad (aided)**	**Rad + AI in I/U**	**Rad + AI (union)**	**AI**
**Reader1**	48.78 (38.0–59.6)	48.81 (38.0–59.6)	65.57 (55.3–75.9)	40 (29.4–50.6)	29.78 (19.9–39.7)
**Reader2**	44.68 (34.6–54.7)	43.88 (33.8–53.9)	58.33 (48.4–68.3)	40.16 (30.2–50.1)	29.7 (20.5–38.9)
**Reader3**	34.38 (26.2–42.6)	34.59 (26.3–42.8)	45 (36.4–53.6)	35.57 (27.3–43.9)	29.17 (21.3–37.0)
**Reader4**	36.61 (27.7–45.5)	36.29 (27.4–45.2)	41.38 (32.3–50.5)	31.65 (23.0–40.3)	28.99 (20.6–37.4)
**All**	40.14 (35.4–44.9)	39.86 (35.2–44.6)	50.14 (45.3–54.9)	36.56 (31.9–41.2)	29.41 (25.0–33.8)
**P-value (Z)**	-	0.1613	0	0.0189	0.0001
**P-value (T)**	-	0.1978	0.0104	0.0647	0.0176

Supplementary Table S8. Per-reader, per-scenario NPVs with 95% confidence intervals, alongside paired z-tests and paired t-tests to assess the significance of results compared to the baseline of unassisted radiologists.

NPV	Rad (unaided)	Rad (aided)	Rad + AI in I/U	Rad + AI (union)	AI
**Reader1**	95.35 (93.5–97.2)	95.54 (93.7–97.4)	95.55 (93.7–97.4)	98.1 (96.9–99.3)	97.61 (96.2–99.0)
**Reader2**	95.64 (93.8–97.5)	95.82 (94.0–97.7)	95.84 (94.0–97.7)	97.42 (96.0–98.9)	96.65 (95.0–98.3)
**Reader3**	95.65 (93.7–97.6)	96.09 (94.2–98.0)	96.15 (94.3–98.0)	97.71 (96.3–99.2)	96.52 (94.8–98.3)
**Reader4**	95.23 (93.2–97.2)	96.03 (94.2–97.9)	96.79 (95.1–98.4)	96.95 (95.3–98.6)	96.61 (94.9–98.3)
**All**	95.46 (94.5–96.4)	95.86 (94.9–96.8)	96.06 (95.2–97.0)	97.55 (96.8–98.3)	96.85 (96.0–97.7)
**P-value (Z)**	-	0.0028	0.0284	0	0
**P-value (T)**	-	0.0349	0.0764	0.0015	0.0108

Supplementary Table S9. Per-reader, per-scenario Recall Rates with 95% confidence intervals, alongside paired z-tests and paired t-tests to assess the significance of results compared to the baseline of unassisted radiologists.

Recall Rate	Rad (unaided)	Rad (aided)	Rad + AI in I/U	Rad + AI (union)	AI
**Reader1**	14.77 (11.8–17.7)	15.14 (12.2–18.1)	10.99 (8.4–13.6)	24.32 (20.8–27.9)	32.07 (28.2–36.0)
**Reader2**	17 (13.9–20.1)	17.72 (14.5–20.9)	13.02 (10.2–15.8)	22.97 (19.5–26.5)	29.84 (26.0–33.7)
**Reader3**	23.62 (20.0–27.2)	24.54 (20.9–28.2)	18.45 (15.2–21.7)	27.49 (23.7–31.2)	31 (27.1–34.9)
**Reader4**	20.29 (16.9–23.6)	22.46 (19.0–25.9)	21.01 (17.6–24.4)	28.62 (24.8–32.4)	30.62 (26.8–34.5)
**All**	18.89 (17.3–20.5)	19.94 (18.3–21.6)	15.85 (14.3–17.4)	25.84 (24.0–27.7)	30.88 (29.0–32.8)
**P-value (Z)**	-	0.0038	0.0092	0	0
**P-value (T)**	-	0.0379	0.0498	0.0059	0.0054

Supplementary Table S10. Checklist for Artificial Intelligence in Medical Imaging (CLAIM): 2024 Update

Section / Topic	No.	Item	Page / Line	No	NA
TITLE / ABSTRACT					
	**1**	Identification as a study of AI methodology, specifying the category of technology used (e.g., deep learning)	**P1**		
ABSTRACT					
	**2**	Summary of study design, methods, results, and conclusions	**P1**		
INTRODUCTION					
	**3**	Scientific and/or clinical background, including the intended use and role of the AI approach	**P3**		
	**4**	Study aims, objectives, and hypotheses	**P3**		
METHODS					
Study Design	**5**	Prospective or retrospective study	**P1, line6+P2, line 8+P4, line3**		
	**6**	Study goal	**P4, line 8**		
Data	**7**	Data sources	**P4, line 10**		
	**8**	Inclusion and exclusion criteria	**P4, line 12**		
	**9**	Data pre-processing	**P5, line 19**		
	**10**	Selection of data subsets	**P4, line 20**		
	**11**	De-identification methods	**P5, line 22**		
	**12**	How missing data were handled			**x**
	**13**	Image acquisition protocol	**P5, line 20**		
Reference Standard	**14**	Definition of method(s) used to obtain reference standard	**P4, line 11**		
	**15**	Rationale for choosing the reference standard		**x**	
	**16**	Source of reference standard annotations	**P5, line 22**		
	**17**	Annotation of test set	**P4, line 11**		
	**18**	Measures of inter- and intra-rater variability of features described by the annotators			**x**
Data Partitions	**19**	How data were assigned to partitions	**P5, line 2**		
	**20**	Level at which partitions are disjoint	**P5, line 2**		
Testing Data	**21**	Intended sample size	**P4, line 27**		

Section / Topic	No.	Item	Page / Line	No	NA
Model	**22**	Detailed description of model	**P5, line 27**		
	**23**	Software libraries, frameworks, and packages		**x**	
	**24**	Initialization of model parameters		**x**	
Training	**25**	Details of training approach	**P6, line 11**		
	**26**	Method of selecting the final model	**P8, line 4**		
	**27**	Ensembling techniques		**x**	
Evaluation	**28**	Metrics of model performance	**P7, line 4**		
	**29**	Statistical measures of significance and uncertainty	**P9, line 3**		
	**30**	Robustness or sensitivity analysis	**P9, line 3**		
	**31**	Methods for explainability or interpretability			**x**
	**32**	Evaluation on internal data	**P9, line 27**		
	**33**	Testing on external data	**P4, line 16**		
	**34**	Clinical trial registration	**P4, line 4**		
RESULTS					
Data	**35**	Numbers of patients or examinations included and excluded	**P9, line 10**		
	**36**	Demographic and clinical characteristics of cases in each partition	**P9, line 14**		
Model performance	**37**	Performance metrics and measures of statistical uncertainty	**P9, line 19**		
	**38**	Estimates of diagnostic performance and their precision	**P9, line 19**		
	**39**	Failure analysis of incorrect results			**x**
DISCUSSION					
	**40**	Study limitations	**P13, line 1**		
	**41**	Implications for practice, including intended use and/or clinical role	**P11, line 23**		
OTHER INFORMATION					
	**42**	Provide a reference to the full study protocol or to additional technical details		**x**	
	**43**	Statement about the availability of software, trained model, and/or data	**Yes**		
	**44**	Sources of funding and other support; role of funders	**Yes**		

* Indicate page and/or line number for each checklist item that is present. NA = not applicable.
